# Influence of Genetic and Epigenetic Factors of P2Y_12_ Receptor on the Safety and Efficacy of Antiplatelet Drugs

**DOI:** 10.1007/s10557-022-07370-8

**Published:** 2022-08-09

**Authors:** Dorota Danielak, Kornel Pawlak, Franciszek Główka, Marta Karaźniewicz-Łada

**Affiliations:** Department of Physical Pharmacy and Pharmacokinetics, Poznan University of Medical Sciences, Rokietnicka 3 St, 60-806 Poznań, Poland

**Keywords:** Platelet aggregation inhibitors, Purinergic P2Y receptor antagonists, Polymorphism, Genetic, Clopidogrel

## Abstract

**Purpose:**

P2Y_12_ receptor inhibitors are drugs that decrease the risk of stent thrombosis and lower the long-term risk of non-stent-related myocardial infarction and stroke. They inhibit the binding of adenosine diphosphate (ADP) to the P2Y_12_ receptor and effectively reduce platelet reactivity. However, considerable variability in the pharmacodynamics response contributes to a failure of antiplatelet therapy; this phenomenon is especially notorious for older drugs, such as clopidogrel. Some genetic polymorphisms associated with these drugs’ metabolic pathway, especially in the CYP2C19 gene, can significantly decrease antiplatelet efficacy. There are few reports on the variability stemming from the target of this drug class that is the P2Y_12_ receptor itself.

**Results and conclusion:**

This review summarizes the results of research that focus on the influence of *P2Y*_*12*_ genetic polymorphisms on the pharmacodynamics and the efficacy of P2Y_12_ inhibitors. We found that the conclusions of the studies are unequivocal, and despite several strong candidates, such as G52T (rs6809699) or T744C (rs2046934), they may not be independent predictors of the inadequate response to the drug. Most probably, P2Y_12_ genetic polymorphisms contribute to the effect exerted by other gene variants (such as CYP2C19*2/*3/*17), drug interactions, or patient habits, such as smoking. Also, epigenetic modifications, such as methylation or miRNA levels, may play a role in the efficacy of antiplatelet treatment.

## Introduction

**Fig. 1:**
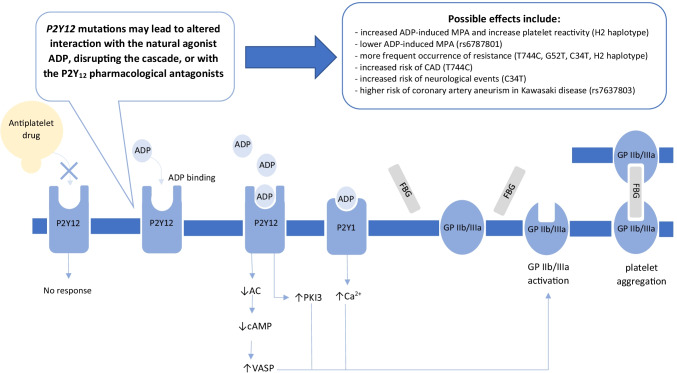
Activation mechanism of P2Y_12_ receptor through agonist. ADP, adenosine diphosphate; AC, adenyl cyclase; CAD, coronary artery disease; cAMP, cyclic adenosine monophosphate; FBG, fibrinogen; MPA, maximal platelet aggregation; PKI 3, phosphoinositide 3-kinase; VASP, vasodilator-stimulated phosphoprotein

Combined with aspirin, P2Y_12_ receptor inhibitors are used in dual antiplatelet therapy (DAPT). This treatment aims to prevent thrombotic events after percutaneous coronary intervention (PCI) or myocardial infarction (MI) and stroke [1]. The basis of the therapeutic action of this drug class is the inhibition of adenosine diphosphate (ADP) binding to the P2Y_12_ receptor. Two platelet receptors, P2Y_1_ and P2Y_12_, react to ADP in physiological conditions and induce platelet aggregation [2]. The introduction of P2Y_12_ inhibitors attenuates ADP interaction with its platelet receptor and effectively reduces platelet reactivity (Fig. 1).

Besides the most widely used clopidogrel, newer P2Y_12_ inhibitors gain interest, and their use increases (Table 1). For example, Chest Pain-Myocardial Infarction Registry in the United States noted that ticagrelor use in patients with ST-segment-elevation myocardial infarction (STEMI) increased from 18.0 to 44.0% [3]. Last year, selatogrel (ACT-246475), a novel subcutaneous P2Y_12_ inhibitor, completed phase 2 studies [4, 5]. The declining use of clopidogrel stems from a sizeable observed variability in the therapeutic response. The “clopidogrel resistance” phenomenon can affect as much as 16–50% of the population [6], leading to the failure of antiplatelet therapy. Significant contributors to this resistance are genetic polymorphisms of CYP450 enzymes, especially CYP2C19 [7]. The presence of loss-of-function CYP2C19 alleles increases the risk of major adverse cardiovascular events (MACE) during DAPT with clopidogrel [8–11]. However, some authors claim that alterations in the P2Y_12_ gene could also play a role in thrombotic events during antiplatelet therapy (Tables 2 and 3) [12, 13]. As these polymorphisms may affect the receptor’s functionality, its potential influence may translate to other P2Y_12_ inhibitors. Therefore, this narrative review focuses on presenting available data on the influence of P2Y_12_ genetic polymorphism on the efficacy and safety of currently used antiplatelet drugs.

**Table 1:** Antiplatelet drugs’ characterization

Drug	Administration route	Metabolic activation	Activating enzymes and reaction type	Inhibition	Reference
Ticlopidine	Oral	+	CYP 2B6, 2C19**,** 1A2, 3A4 (oxidation)	Irreversible	[14, 15]
Clopidogrel	Oral	+	CYP 2C19, 1A2, 2B6, 3A4 (oxidation)	Irreversible	[15–18]
Prasugrel	Oral	+	Esterases (hydrolysis), CYP 3A5, 2B6, 2C9 and 2C19 (oxidation)	Irreversible	[15, 19–22]
Vicagrel	Oral	+	Carbohydrate esterase family 2 and paraoxonase 1 (hydrolysis), CYP 2C19, 3A4, 2B6 (oxidation)	Irreversible	[23, 24]
Selatogrel	Subcutaneous	-	-	Reversible	[25, 26]
Elinogrel	Oral/intravenous	-	-	Reversible	[27, 28]
Ticagrelor*	Oral	±	CYP 3A4 (oxidation)	Reversible	[29, 30]
Cangrelor	Bolus or intravenous infusion	-	-	Reversible	[31]

^*^Parent compound is pharmacologically active

**Table 2:** Genetic polymorphisms in the P2Y12 gene sequence with possible influence on the efficacy of antithrombotic drugs. Data taken from the dbSNP database (ncbi.nlm.nih.gov/snp/). Global allele frequencies (AF) taken from the Ensembl database, 1000 Genomes Project Phase 3 (ensembl.org). The alleles leading to a possible function alteration are in bold

Accession number	Position	Common name	Region	Change	AF	Modification	Function alteration
rs6798347	g.1387C > T	-	Promoter	G > A	G: 0.711 **A: 0.289**	Promoter modification	Possible lower incidence of HTPR (as part of H1 haplotype) [32]
rs1907637	g.2707 T > C	-	Promoter	G > A	G: 0.903 **A: 0.097**	Promoter modification	May influence baseline platelet aggregation [33]
rs6787801	g.7804 T > G	-	Intron	T > C	T: 0.527 **G: 0.473**	Intron variant	Possible lower incidence of HTPR (as part of H1 haplotype) [32]
rs9859552	g.16549C > T	-	Intron	G > T	G: 0.938 **T: 0.062**	Intron variant	Noticeable but not significantly greater platelet reactivity [34]
rs3732759	g.32620 T > C	-	Intron	A > G	A: 0.678 **G: 0.322**	Intron variant	Occurs more frequently in patients with CVD
rs7428575	g.32922A > C	-	Intron	T > G	**T: 0.678** G: 0.322	Intron variant	Increased risk of CHD [35]
rs6801273	g.44715A > G	-	Intron	T > C	T: 0.583 **C: 0.417**	Intron variant	Possible lower incidence of HTPR (as part of H1 haplotype) [32]
rs10935838	g.49298 T > C	i-C139T	Intron	C > T	C: 0.868 **A: 0.132**	Intron variant	Presence increases maximum platelet aggregation and reduction in cAMP (in H2 haplotype) [13]
rs2046934	g.49903C > T	i-T744C / T744C	Intron	T > C	T: 0.868 **C: 0.132**	Intron variant	Possible lower inhibition of platelet aggregation, higher peak platelet aggregation, elevated platelet reactivity [36, 37]
rs5853517	g.49960del	i-ins801A	Intron	Ins A	A: 0.868 **AA: 0.132**	Intron variant, insertion/ deletion	Presence increases maximum platelet aggregation and reduction in cAMP (in H2 haplotype) [13]
rs6785930	g.50929C > T	C34T	Exon 2 (short transcript)	C > T	C: 0.758 **T: 0.242**	Missense (Asn > Lys)	Lower incidence of HTPR (as part of H1 haplotype) [32]
rs6809699	g.50947 T > G	G52T	Exon 2 (short transcript)	G > T	G: 0.912 **T: 0.088**	Synonymous (Gly > Gly)	Presence increases maximum platelet aggregation and reduction in cAMP (in H2 haplotype) [13]

*CVD* cardiovascular disease, *HTPR* high on-treatment platelet reactivity

**Table 3:** The influence of chosen genetic polymorphism on the platelet reactivity and the efficacy of the antiplatelet treatment

Reference	Studied P2Y12 SNPs	Study group	Drug tested	Observed effects	Pharmacodynamic assessment	Results
In vitro assessment only						
[13]	T744C, C139T, 801A, G52T, C34T	98 healthy volunteers (Caucasian origin)	None (ADP reactivity test only)	Assessment of maximal platelet aggregation between P2Y12 genotypes and haplotypes	Photometric method	The H2 haplotype was related to increased maximal platelet aggregation after ADP exposition
[38]	T744C, C139T, 801A, G52T, C34T	158 healthy volunteers (of Korean origin)	None (ADP reactivity test only)	Assessment of the relationship between ADP-induced MPA and studied SNPs	LTA/platelet-rich plasma turbidimetry	ADP-induced maximal platelet aggregation was not affected by three intronic P2Y12 and C34T polymorphisms. However, the authors noticed a correlation between the TT genotype of G52T polymorphism and higher ADP-induced maximal platelet aggregation
[33]	T744C, G52T, C34T, rs1907637, rs79320243, rs10935842, rs6787801, rs6801273, rs16863323	196 healthy volunteers (Chinese origin)	Ticagrelor, in vitro test only	Assessment of MPA and relative inhibition of platelet aggregation	LTA/platelet-rich plasma turbidimetry	Any genetic variations both in the P2Y12 and G-protein beta 3 subunit were not related to differences in platelet inhibition after partial ex vivo blockade using ticagrelor. Several P2Y12 polymorphisms and one haplotype (haplotype D) were related to differences in baseline platelet aggregation, but no studied polymorphism affected response to ticagrelor in the ex vivo study
[36]	T744C, rs6798347, rs6787801, rs9859552, rs6801273	242 healthy volunteers (mostly of Caucasian origin)	Cangrelor, in vitro test only	Assessment of MPA and relative inhibition of platelet aggregation	LTA/platelet-rich plasma turbidimetry	The minor C allele of rs6787801 was related to lower ADP-induced maximal platelet aggregation. rs9859552 AA genotype was related to decreased response to cangrelor therapy when compared with CC genotype. Haplotypes analysis presented results similar to the results of SNPs
[37]	T744C, C34T	29 healthy volunteers (mostly of Caucasian origin)	Cangrelor, in vitro test only	Assessment of TRAP-mediated platelet activation by PAC1 binding and CD62P expression, and relative inhibition of platelet aggregation	Flow cytometry, VerifyNow P2Y12 assay	Patients with H2/H2 haplotypes showed greater platelet inhibition during cangrelor exposition. No consistent effects of the C34T and T744C polymorphisms separately were found
Influence of studied P2Y12 SNPs on platelet reactivity or clinical outcome						
[39]	T744C	77 ACS patients & 101 healthy volunteers (of Moroccan origin)	Clopidogrel, 300 mg LD & 75 mg MD	Association between genotypes and ACS risk and CR, defined as 208 PRU and above or < 20% inhibition of platelet aggregation after 7 days of treatment	VerifyNow P2Y12 assay	The C allele of studied polymorphism was more frequent among patients resistant to therapy. Relation of C allele to observed ACS was statistically significant
[35]	T744C, rs7428575, rs3732759	178 CHD patients and 182 healthy controls (Chinese origin)	Clopidogrel, 300 mg LD & 75 mg MD	Assessment of the decrease in MPA. CR defined as a < 10% decrease from baseline after 10 days of treatment	LTA/platelet-rich plasma turbidimetry	Significant differences in genotype and allele frequencies of T744C and rs3732759 between the case and control groups were observed. Haplotype TCA was related to increased coronary artery disease risk. Responsive to clopidogrel therapy group consisted of coronary artery disease patients with higher frequencies of TT genotype of T744C and lower frequencies of GG genotype of rs3732759 compared to patients with CR
[40]	T744C	40 ACS patients and 40 age-matched healthy controls (origin unspecified)	Clopidogrel; exact dosing regimen unspecified	CR defined as persistence of HPR (ADP-Ag > 70%) Comparison of allele frequencies between patients and controls; prevalence of CR according to the genotype C	LTA/platelet-rich plasma turbidimetry	Carriers of T744C C allele showed increased platelet reactivity after ADP activation
[41]	T744C	191 patients with ischemic stroke (Chinese origin)	Clopidogrel 300 mg LD & 75 mg MD	CR defined as a < 10% decrease in MPA after 5 days of treatment	LTA/platelet-rich plasma turbidimetry	The C allele in T744C P2RY12 polymorphism was considered to decrease the risk of CR. Additionally, CR was found to be related to increased risk of hypertension
[42]	T744C	268 patients with ischemic stroke (Chinese origin)	Clopidogrel, 75 mg	Primary endpoints: transient ischemic attack, ischemic stroke, myocardial infarction or vascular-related mortality	-	There was significant association between A allele of the T744C P2Y12 polymorphism and present adverse effects
[32]	T744C, C34T, rs6798347, rs6787801, rs6801273	180 patients with ACS (Chinese origin)	Clopidogrel, 75 mg MD, with or without 300/600 mg LD	HTPR defined by platelet inhibition > 30%	Thromboelastography	Authors determined six haplotypes on basis of the selected P2RY12 and CYP2C19 polymorphisms (named as H_0_—H_5_). They discovered, that combination of few P2RY12 variations rather than T744C alone, associated with different response to treatment with clopidogrel
[43]	T744C, G52T, C34T	146 patients with CAD (Han Chinese origin)	Clopidogrel, 75 mg MD	HTPR defined by MPA > = 50%	LTA/platelet-rich plasma turbidimetry	There was no association between studied polymorphism and HTPR or recurrence of major adverse cardiac events
[44]	T744C, C34T	124 patients with acute myocardial infarction (Caucasian origin)	Clopidogrel, 75 mg MD	CR defined as values > 45% in LTA or 298 AUC/min in Multiplate	LTA/platelet-rich plasma turbidimetry and Multiplate analyzer	There was not any statistically significant association between P2RY12 receptor polymorphisms (T744C, C34T) and response to antiplatelet therapy with clopidogrel
[45]	T744C	60 patients with ACS (Mexican origin)	Clopidogrel 300 mg LD & 75 mg MD	CR defined as persistence of HPR (ADP-Ag > 70%)	LTA/platelet-rich plasma turbidimetry	T744C polymorphism showed no association with clopidogrel resistance
[46]	T744C	100 patients with CAD and ACS or chronic stable angina (North Indian origin)	Clopidogrel 75 mg MD, 300 mg LD in one subject	Assessment of the decrease in MPA. CR defined as a < 10% decrease from baseline, semi-responders as 10–29% reduction, responders as > 30% reduction	LTA/platelet-rich plasma turbidimetry	H1/H1 haplotype (P2Y12 polymorphism) was related to CR. CR was not related to single ADP receptor P2Y1 and P2Y12 gene polymorphisms
[47]	T744C	1419 ACS patients (origin unspecified)	Clopidogrel 600 mg LD & 75 mg MD	CR defined as persistence of HPR (ADP-Ag > 70% or Arachidonic acid-AG > 20%) after 6 days	LTA/platelet-rich plasma turbidimetry	T744C polymorphism was not associated with higher platelet reactivity
[48]	T744C	597 patients with ACS (origin unspecified)	Clopidogrel 600 mg LD	CR defined as persistence of HPR (ADP-Ag > 70%) after LD; also VASP value and P-selectin surface expression	LTA/platelet-rich plasma turbidimetry, VASP phosphorylation, flow cytometry	T774C polymorphism was not associated with CR
[49]	T744C	120 patients scheduled for elective PCI (mixed origin)	Clopidogrel 300 mg LD & 75 mg MD	CR defined as a < 10% decrease from baseline after 3 months	LTA/platelet-rich plasma turbidimetry, flow cytometry	No divergence in polymorphism frequencies between resistant and nonresistant patients was observed. No significant differences were observed also in response to aspirin or to clopidogrel in patients grouped accordingly to their genotype
[50]	T744C	119 patients with CAD (origin unspecified)	Clopidogrel 300 mg LD & 75 mg MD or 75 mg MD only	MPA assessment and platelet activation with fibrinogen, and P-selectin expression	LTA/platelet-rich plasma turbidimetry, flow cytometry	T744C polymorphism was not related to differences in response to clopidogrel at early or late stage of therapy
[51]	T744C, C34T	222 patients with ACS (Chinese Han origin)	Clopidogrel 300 mg LD & 75 mg MD	HPR cut-off point has been described as ≥ 208 PRU	VerifyNow P2Y12 assay	Poor response to clopidogrel was based on coexistence of CYP2B6*9 and T744C and coexistence of CYP2B6*1B and T744C
[52]	G52T, C34T	503 STEMI patients (Chinese Han origin)	Clopidogrel 300 mg LD & 75 mg MD	Primary endpoint was a composite of cardiovascular death, nonfatal MI, vessel revascularization, and S. Safety endpoint was incidence of major bleeding events	-	G52T minor allele was factor responsible for major bleedings
[53]	G52T, C34T	473 patients with PAD	Clopidogrel, 75 mg (in 137 patients)	Primary endpoint of neurological events (ischemic stroke, carotid endartectomy, carotid stenting)	-	Clopidogrel treated patients with at least one C34T T allele showed 4.02-fold higher risk for neurological events comparing to carriers of only C34T C alleles. None of the studied polymorphisms was related to all-cause mortality
[54]	G52T, C34T	498 patients with ACS	Clopidogrel 300 mg LD & 75 mg MD	CR defined as < 10% decrease from baseline after 7 days of treatment. Secondary outcome comprised adverse cardiovascular events	AggRAM system	Patients with T allele at G52T or C34T polymorphisms, presented significantly higher risk of CR* and presence of cardiovascular events in comparison to wild-type patients. Patients with the T variations in C34T presented also significantly increased risk of post percutaneous coronary intervention and higher recurrence risk of cardiovascular diseases
[55]	G52T	557 patients with CAD and 43 healthy volunteers (origin unspecified)	Clopidogrel 300, 450 or 600 mg LD & 75 mg MD (in patients only)	Nonresponsiveness defined as impedance above 5 Ω	Impedance aggregometry	Patients with H2/H2 haplotype were more often nonresponders and showed stronger platelet aggregation than subjects with at least one H1. Haplotype H2/H2 was also related with increased CR
[56]	T744C	26 patients with acute MI and 102 healthy volunteers (Western Indian origin)	Clopidogrel 300 mg LD & 75 mg MD	Assessment of MPA and inhibition of platelet aggregation before, 24 h and 6 days of treatment	LTA/platelet-rich plasma turbidimetry	Beside T744C P2Y12 polymorphism MDR1 and CYP2C19 SNPs were taken into consideration. T744C and CYP2C19*2 mutant alleles were related to clopidogrel resistance, compared to wild type
[57]	T744C	112 patients scheduled for PCI (Iranian origin)	Clopidogrel 600 mg LD & 150 and 75 mg MD	Responsiveness to clopidogrel defined a decrease from baseline value; < 10% for nonresponders, 10 – 30% for semiresponders, > 30% for responders	LTA/platelet-rich plasma turbidimetry	No significant association between response to clopidogrel treatment and P2Y12 polymorphism was observed
[56]	T744C	26 patients with acute MI and 102 healthy volunteers (Western Indian origin)	Clopidogrel 300 mg LD & 75 mg MD	Assessment of MPA and inhibition of platelet aggregation before, 24 h and 6 days of treatment	LTA/platelet-rich plasma turbidimetry	Beside T744C P2Y12 polymorphism MDR1 and CYP2C19 SNPs were taken into consideration. T744C and CYP2C19*2 mutant alleles were related to clopidogrel resistance, compared to wild type
Comparison of P2Y12 SNP prevalence in study populations						
[58]	T744C, C34T	696 patients with CAD (Caucasian origin)	Unspecified	Assessment of bleeding complications defined as major bleeding, intercranial bleeding, or clinically over bleeding, minor bleeding relative to studied polymorphisms	-	Studied polymorphisms were related to bleeding incidents
[59]	T744C, C34T	105 patients with premature MI, and 132 patients without CAD (Caucasian origin)	Unspecified	Assessment of SNP frequencies in study groups and evaluation of cardiovascular risk factors	-	No effect of P2Y12 polymorphism was observed
[60]	T744C, G52T, rs10935838 rs5853517	A total of 1982 patients and matched controls: with/without MI, with/without transient ischemic attacks, with/without deep venous thrombosis (mostly Caucasian origin)	Unspecified	Comparison of SNPs prevalence between cases and controls	-	Haplotype H2 was significantly related to lower risk of incident deep vein thrombosis and pulmonary embolism, compared to haplotype H1. There was no association between the P2RY12 polymorphisms or the H2 haplotype with possible myocardial infarction or ischemic stroke
[61]	T744C, G52T, rs9859538, rs1491974, rs7637803	776 patients with Kawasaki disease and 1335 healthy controls (Southern Chinese origin)	-	Assessment of P2Y12 SNP association with coronary aneurism in Kawasaki disease	-	No significant association between any of studied polymorphism and Kawasaki disease was found. There was however significant association between TT genotype of rs7637803 and higher risk of coronary artery aneurysm risk in Kawasaki disease patients. Authors claimed that it could be used as biomarker for prediction of giant coronary artery aneurysm
[62]	T744C, G52T, rs9859538, rs1491974, rs7637803	759 patients with Kawasaki disease (Chinese Han origin)	-	Association of studied SNPs with intravenous immunoglobulin resistance	-	After including such factors as gender and age, carriers of the rs6809699 (G52T) C allele were at lower risk of intravenous immunoglobulin resistance. There was no significant association between four other polymorphism and sensitivity to intravenous immunoglobulin
[63]	T744C, G52T, rs6801273, rs6798347	122 patients with MI (subdivided according to the age when MI occurred) and 235 healthy controls	Unspecified	Assessment of studied SNPs prevalence between study groups	-	Studied P2RY12 polymorphisms did not show any significant correlations to CR

*ACS* acute coronary syndrome, *CHD* coronary heart disease, *CR* clopidogrel resistance, *HTPR* high on-treatment platelet reactivity, *LD* loading dose, *LTA* light transmittance aggregometry, *MD* maintenance dose, *MPA* maximum platelet aggregation rate, *PAD* peripheral artery disease, *PCI* percutaneous coronary intervention, *STEMI* ST-elevation myocardial infarction, *ST* stent thrombosis

## H1/H2 Haplotype and Related Alleles

In 2003, Fontana et al. [13] identified that some healthy volunteers had significantly greater ADP-induced maximal platelet aggregation (MPA). The authors performed genotyping and found that four of the studied polymorphisms (G52T (rs6809699), i-C139T (rs10935838), i-T744C (rs2046934), and i-ins801A (rs5853517); Table 2) were in linkage disequilibrium. When a subject carried G variant in G52T, C in i-C139T, T in i-T744C, and lacked insertion in 801A, he carried H1 haplotype. Otherwise, an H2 haplotype was present. P2Y12 genotyping revealed that greater MPA and a more significant reduction in cAMP concentration correlated with the presence of H2 haplotype.

Further investigations showed that the H2 haplotype could be related to thrombotic-related diseases. For example, a case–control study showed that patients with peripheral arterial disease are more likely to be H2 carriers (30% in cases vs. 21% in healthy controls) [12]. Likewise, a recent China-based study revealed that 26.3% of subjects with cerebral infarction carried H2 haplotype, while this variant occurred less frequently (16.7%) in healthy controls [64]. The most widely used allele indicative of the H1/H2 haplotype is the intronic variant T744C. Several case–control studies conducted on patients with acute coronary syndrome (ACS) or coronary artery disease observed that the mutant C allele was more frequent in the ACS group: 22.73% vs. 19.13% [39], 25.4% vs. 31.5% [35], and 27.5% vs. 2.5% [40] in cases and controls, respectively. These findings confirm that H2 polymorphisms can be connected with cardiovascular diseases and influence platelet reactivity. Consequently, they may alter the efficacy of the antiplatelet treatment with P2Y_12_ inhibitors.

### H1/H2 Haplotype Alleles and Clopidogrel

Two meta-analyses investigated the association of P2Y_12_ single nucleotide polymorphisms (SNPs) linked with H1/H2 haplotypes and the efficacy of clopidogrel. The first one focused on the risk of clopidogrel resistance, defined either as lower than 10% platelet inhibition or ADP-induced platelet aggregation greater than 70% [65]. The authors found that under the dominant model, only G52T increased the odds ratio of clopidogrel resistance (OR = 1.45, 95% CI: 1.14–1.85, *p* = 0.003 for G52T). The other investigated SNP, T744C, did not influence the odds of inadequate response to clopidogrel. The second meta-analysis, by Zhao et al. [66], showed a significant association of T744C and ischemic events in the Han Chinese population but not in the Caucasian population. Moreover, the significance was observed only under a recessive model (CC vs. CT + TT, OR = 3.32, 95% CI: 1.62–6.82, *p* = 0.001). In contrast, Liu et al. [41] found that in the case of patients with ischemic stroke, the presence of the C allele was associated with a lower incidence of clopidogrel resistance (OR = 0.407, 95% CI: 0.191–0.867, *p* = 0.018). Another study on the population of Asian patients with symptomatic extracranial or intracranial stenosis showed a significant adverse influence of the T allele. When a patient carried the T allele, there were significantly greater odds of transient ischemic attack, ischemic stroke, MI, or vascular-related mortality during a 1-year follow-up (OR: 2.01, 95% CI: 1.10–3.67, *p* = 0.041) [42].

Despite these reports, a number of studies failed to notice any influence of T744C on the prevalence of HTPR or adverse events [32, 34, 39, 43–50, 58, 67, 68]. It is also possible that the effect of T744C on platelet function and the efficacy of clopidogrel treatment may depend on the coexistence of other factors, such as compliance, drug interactions, and comorbidities [51, 69, 70]. For example, a study on 222 ACS patients showed that the concomitance of a mutant allele of T744C and polymorphic alleles of *CYP2B6* (*9 and *1B) were associated with high platelet reactivity and poor response to clopidogrel [71].

Another SNP associated with H1/H2 haplotype, G52T, can negatively impact PCI outcomes in elderly patients. In a recent study that included 811 patients aged ≥ 75 years of age, TT homozygotes had a higher risk of bleeding during a 1-year follow-up compared with GG and GT genotypes (adjusted HR: 3.87, 95% CI: 1.41–10.68, *p* = 0.009) [72]. Although T744C was not one of the investigated variants, the T allele in G52T is associated with the H2 haplotype. Therefore, the observation of Cha et al. could translate to the combined effect of this haplotype on the efficacy of DAPT with clopidogrel (75 mg) and aspirin (100 mg).

### H1/H2 Haplotype Alleles and Other P2Y_12_ Inhibitors

Available resources regarding the influence of H1/H2 on the efficacy of other P2Y_12_ inhibitors are less abundant than for clopidogrel. One study investigated the role of two alleles comprising H1/H2 haplotypes (T744C and G52T) on ticagrelor’s ex vivo antiplatelet effect [33]. The carriers of the T744C and G52T mutant alleles had significantly lower baseline platelet aggregation. However, there were no statistically significant differences in peak and late aggregation or inhibition of platelet aggregation at low (15 μM) and high (50 μM) ticagrelor concentrations.

In a cangrelor-focused study, Bouman et al. [36] investigated ex vivo peak and late platelet aggregation at low and high drug concentrations. The carriers of the T744C CC genotype exhibited higher peak platelet aggregation and lower peak inhibition of platelet aggregation at low (0.05 µM) cangrelor concentrations but the differences were not statistically significant. In contrast, Oestreich et al. [37] reported a significant recessive effect of H2 haplotype, represented by the T744C variant, on platelet reactivity stimulated by thrombin receptor–activating peptide (TRAP). The response to TRAP was markedly reduced (25–42%) in H2/H2 carriers whose isolated platelets were subjected to cangrelor.

### C34T and P2Y_12_ Inhibitors

Two of the meta-analyses cited above investigated the influence of C34T (rs6785930) on the efficacy of clopidogrel therapy. Both found a significant relationship between the presence of the T allele and the risk of insufficient response to the drug. In the first analysis, the presence of the T allele increased over two-fold the risk of clopidogrel resistance (OR = 2.30, 95% CI: 1.50–3.51, *p* = 0.0001) [65]. In the other study, the odds ratio of ischemic events was 1.7 (95% CI: 1.22–2.36, *p* = 0.002) in carriers of the T allele, but the effect was significant in Han Chinese population only [66]. Also, this polymorphism was not associated with an increased risk of bleeding events.

Lack of the influence of C34T on clopidogrel therapy was reported for European populations [34, 44]. Ulehlova et al. [44] did not observe significant differences in the frequencies of C34T alleles between clopidogrel-resistant patients with AMI and the entire investigated group. This polymorphism also seems not to influence the endothelial function or arterial wall properties [73]. However, an interesting observation was reported for smokers [74]. Carriers of mutant C34T allele with coronary artery disease (CAD) undergoing PCI who also smoked had a higher risk of reaching the primary endpoint (death from cardiovascular causes, nonfatal MI, revascularization, stroke, recurrent cardiac ischemia, or transient ischemic attack) than the CC homozygotes (HR 2.23, 95% CI: 1.05–6.01, *p* = 0.04).

The influence of the C34T allele on the efficacy of ticagrelor may also be insignificant. Although there are no clinical reports, the ex vivo study showed that there might be a difference in platelet aggregation and the inhibition caused by ticagrelor [33]. The CT heterozygotes had significantly lower platelet aggregation and greater inhibition of platelet aggregation at the highest studied ticagrelor concentrations than TT homozygotes.

## Other P2Y12 Haplotypes and Alleles

Besides H1/H2, some reports distinguish other haplotypes that may influence platelet reactivity. Nie et al. [32] investigated SNPs in the promoter and regulatory region of the *P2Y*_*12*_ gene. The authors inferred six commonly occurring haplotypes of four SNPs—rs6798347 (C > t), rs6787801 (T > c), rs6801273 (A > g), and rs6785930 (C > t). Among the haplotypes labeled H0–H5, haplotype H1 (tcgt) had a lower incidence of high-on-treatment platelet reactivity (HTPR) in all of the 180 patients with ACS compared with the reference H0 (CTAC) haplotype (adjusted OR 0.13, 95% CI: 0.03–0.68, *p* = 0.016). The influence was significant after adjusting for other covariates—patients’ demographics and *CYP2C19* LoF alleles.

In a study with patients with ischemic stroke who underwent a stenting procedure, two P2Y_12_ SNPs, rs6787801 and rs6798347, comprised three haplotypes [42]. The patients were on DAPT with clopidogrel and aspirin and were monitored for the occurrence of ischemic events for 12 months after the procedure. None of the distinguished haplotypes was associated with a greater risk of subsequent vascular events.

Li et al. [33] distinguished six common haplotypes (A–F) from nine investigated *P2Y*_*12*_ SNPs. One of the haplotypes (‘D’, TAATAGCCT) included, among others, the minor C allele of T744C, the major C allele of C34T, and the minor T allele of G52T. The carriers of this haplotype exhibited significantly lower baseline platelet aggregation than the most common haplotype. However, it did not translate to significantly lower aggregation or lower inhibition of platelet reactivity caused by ticagrelor. Hence, the authors concluded that the investigated *P2Y*_*12*_ haplotypes do not influence the antiplatelet effect of the drug.

Bouman et al. [36] distinguished six common haplotypes (A–F) from five SNPs in the *P2Y*_*12*_ gene. The studies SNPs were rs6798347, rs6787801, rs9859552, rs6801273, and T744C (rs2046934). The authors found that the C haplotype, comprising the minor T744C C allele and the major variants of the other SNPs, had the most significant influence on cangrelor-induced platelet aggregation. Both peak and late ex vivo platelet aggregation were greater than the reference haplotype under low and high cangrelor concentrations. At the same time, the C haplotype had lower inhibition of platelet aggregation, but the effect was most pronounced 360 s after stimulation with ADP.

Another study showed that the minor G of rs3732759 (A > G) allele occurred more frequently in patients with cardiovascular diseases treated with clopidogrel than in healthy controls (42.7% vs. 26.7%, OR = 1.43, 95% CI: 1.05–1.93, *p* = 0.021) [35]. This observation was also confirmed for the frequency of clopidogrel resistance, as 54.2% of the resistant and only 28.6% of sensitive patients carried GG genotype (*p* = 0.017). Decreased sensitivity to clopidogrel translated to the occurrence of MACE. The patients with MACE were more likely to carry the rs3732759 GG genotype. The authors paired three analyzed SNPs into four common haplotypes in the same paper. It allowed capturing the interplay between the SNPs. Although the rs3732759 GG negatively influenced clopidogrel therapy, the only haplotype associated with an increased risk of CHD was TCA (rs7428575 T, rs2046934 C, and rs3732759 A; OR: 1.57, 95% CI: 1.14–2.17, *p* = 0.005).

Recently, Rath et al. [34] investigated patients with 103 ischemic stroke or transient ischemic attack and investigated the influence of selected SNPs on the platelet reactivity and the degree of aggregation inhibition during clopidogrel therapy. The minor T allele (rs9859552) carriers appeared to have greater platelet reactivity values; the effect was not statistically significant.

## Epigenetic Studies on P2Y12

Epigenetic studies are a new direction to identify the mechanism of variable response to antiplatelet therapy. Several reports suggest that methylations of promoters or other epigenetic changes contribute to the altered expression of *P2Y*_*12*_ and could be responsible for the epigenetic mechanisms of clopidogrel resistance. Su et al. [75] reported that DNA methylation levels of two cytosine-phosphate-guanine (CpG) dinucleotides on *P2Y*_*12*_ promoter (CpG1 and CpG2) were related to platelet activity measured by the VerifyNow *P2Y*_*12*_ assay in patients with CAD. Lower methylation of two CpGs indicated the poorer clopidogrel response in alcohol-abusing status. Moreover, lower methylation levels of CpG1 correlated with higher platelet activity in the active smokers and patients with albumin concentrations $\le$ 35 g/L. In the study by Li et al. [76], the impact of the *P2Y*_*12*_ promoter DNA methylation on the recurrence of ischemic events was evaluated in patients with cerebrovascular disease. Among sixteen tested CpG dinucleotides, three CpG sites (CpG11 and CpG12 + 13) showed lower methylation levels, which correlated with ADP inhibition rate and ADP-induced platelet–fibrin cloth strength measured by thromboelastography. The lower methylation status of CpG11 and CpG12 + 13 was also associated with an increased risk of clinical events, including vascular-related mortality, ischemic stroke, transient ischemic attack, or MI.

Evidence suggests that platelet-related miRNAs could play a pivotal role as biomarkers of antiplatelet therapy efficacy because they act as modulators of *P2Y*_*12*_ expression. miRNAs are small non-coding sequences of nucleotides that bind to mRNA sites, block transcription, and, in consequence, cause a decrease in protein production [77]. Landry et al. [78] reported that the P2Y_12_ receptor is a target of miRNA-223, and complexes of Argonaute 2 protein with miRNA-223 may be involved in regulating P2Y_12_ receptor expression in platelets. These results seem to align with the study by Chyrchel et al. [79], who reported that miRNA-223 expression in plasma was elevated in patients with ACS exhibiting increased platelet inhibition in response to DAPT with aspirin plus clopidogrel, prasugrel, or ticagrelor. The effect was stronger for newer P2Y_12_ antagonists. Similarly, an association between decreased platelet miRNA-223 levels and high on-treatment platelet reactivity in patients treated with clopidogrel was confirmed in other studies [80, 81]. Alteration in platelet aggregation due to reduced P2Y_12_ expression caused by miRNA-126 inhibition was confirmed in patients with ACS [82]. In another study on that group of patients, the statistically significant connection between miRNA-29 and miRNA-34 expression levels and P2Y_12R_ gene polymorphism (A > G, rs3732759) was reported [83]. Syam et al. [84] noticed a significant association of high miRNA-26a platelet expression but not DNA methylation of the P2Y_12_ gene on platelet reactivity in patients with acute STEMI undergoing clopidogrel therapy. The role of an elevated miRNA-92a as a biomarker of an increased risk of ACS was confirmed in coronary heart disease patients with type 2 diabetes mellitus [85]. However, as Kramer et al. [86] claimed, further studies are needed to elaborate a standardized protocol for miRNA sample handling to minimize preanalytical variability and the kinetics of platelet miRNA release in response to platelet activation.

## Summary

With an inadequate response to P2Y12 inhibitors, thrombotic events may occur, leading to treatment failure. As shown in Table 3, some authors claim that alterations in the P2Y_12_ gene could also play a role in thrombotic events during antiplatelet therapy. This review outlines that the H2 haplotype could be most strongly related to thrombotic-related diseases and possibly influences platelet reactivity. However, several studies showed that only G52T, one of the polymorphisms comprising H1/H2 haplotypes, increased the odds of clopidogrel resistance, simultaneously exposing the lack of influence of T744C polymorphism. The coexistence of other factors should be considered regarding platelet function and the efficacy of clopidogrel treatment. The non-genetic factors include the origin of study population or smoking status. While for the newer antiplatelet drugs, the reports are scarce and inconclusive, and some clopidogrel co-factors are well described. For example, the two-step activation process involves several CYP450 enzymes, among which CYP2C19 is regarded as the most influential [7, 87]. The carriers of *CYP2C19* alleles are at the greater risk of MACE, MI, or stent thrombosis. The association is so pronounced that some clinicians postulate guided antiplatelet therapy that includes an option of switching from clopidogrel to newer, CYP2C19-independent alternatives [88]. As CYP2C19 is crucial in the second step of the activation, its inhibitors may also decrease clopidogrel’s efficacy. For example, in a recent meta-analysis, it was shown that concomitant intake of proton pump inhibitors increases the risk of MACE by 63% in CYP2C19 loss-of-function allele carriers (95% CI: 1.31–2.03, *p* < 0.0001) [89]. These findings indicate that studies on P2Y_12_ polymorphisms in patients treated with clopidogrel should account for the CYP2C19 genotype.

Recent evidence indicates that epigenetic factors such as P2Y_12_ DNA methylation and miRNAs play a role in the observed variability of platelet reactivity and may serve as biomarkers of response to P2Y_12_ inhibitors. Lower DNA methylation of the P2Y_12_ gene promoter increases the risk of resistance to antiplatelet therapy, while decreased miRNA levels are connected to high on-treatment platelet reactivity and ischemic events in patients treated with P2Y_12_ inhibitors. Due to a broad range of platelet miRNAs, further studies are needed on a reliable miRNA-based biomarker to assess the in vivo platelet activation and response to P2Y_12_ inhibitors.

## Conclusions

The current review underlines the multifactorial causes of the observed response variability to P2Y_12_ receptor inhibitors. Most of the reported P2Y_12_ genetic polymorphisms (e.g., T allele in G52T) may be responsible for changes in the structure of the P2Y12 receptor, therefore decreasing the efficacy of antiplatelet therapy. However, the impact of some factors (e.g., T744C, C34T, smoking) on antiplatelet therapy is not yet fully determined, and their coexistence may lead to resistance to antiplatelet therapy.

## Data Availability

Not applicable.
